# The Effect of glycocholic acid on the growth, membrane permeability, conjugation and antibiotic susceptibility of Enterobacteriaceae

**DOI:** 10.3389/fcimb.2025.1550545

**Published:** 2025-03-20

**Authors:** Bar Piscon, Boris Fichtman, Amnon Harel, Amos Adler, Galia Rahav, Ohad Gal-Mor

**Affiliations:** ^1^ The Infectious Diseases Research Laboratory, Sheba Medical Center, Ramat-Gan, Israel; ^2^ Department of Clinical Microbiology and Immunology, Tel Aviv University, Tel Aviv, Israel; ^3^ Faculty of Medical and Health Sciences, Tel Aviv University, Tel Aviv, Israel; ^4^ Azrieli Faculty of Medicine, Bar-Ilan University, Safed, Israel; ^5^ Clinical Microbiology Laboratory, Tel-Aviv Sourasky Medical Center, Tel-Aviv, Israel

**Keywords:** bacteria, bile, glycocholic acid, conjugation, membrane, antibiotic resistance, Enterobacteriaceae, gram-negative

## Abstract

**Introduction:**

Glycocholic acid (GCA) is a steroid acid and one of the main glycine-conjugated bile components in mammalian bile, which is involved in the emulsification and absorption of fats and sterols. It is long-known that the amphipathic nature of bile acids enables them to interact with the lipid membrane of Gram-positive bacteria and act as potent antimicrobial compounds. Nevertheless, Gram-negative Enterobacteriaceae species inhabiting the intestinal tract of mammals are considered to be more bile-resistant compared to Gram-positive bacteria and are thought to tolerate high bile concentrations.

**Results:**

Here, we show that 1-2% of GCA inhibit the growth of Enterobacteriaceae species, including *E. coli*, *Salmonella enterica*. *Klebsiella* spp., *Citrobacter* spp., and *Raoultella* spp. during their late logarithmic phase in liquid culture, but not in solid media. Despite their lipopolysaccharide membrane layer, we demonstrate that, in liquid, GCA increases permeability, changes the surface of the Enterobacteriaceae membrane, and compromises its integrity. These changes result in leakage of cytoplasmic proteins and enhancement of their susceptibility to antibiotics. Moreover, GCA significantly reduces bacterial motility, the frequency of bacterial conjugation and horizontal acquisition of antibiotic resistance genes. These phenotypes are associated with repression of flagellin (*fliC*) transcription and a sharp decrease in the occurrence of conjugative pili in the presence of glycocholic acid, respectively.

**Discussion:**

Overall, these findings broaden the current understanding about bile resistance of Gram-negative bacteria and suggest that GCA can be used to inhibit bacterial growth, augment the activity of antimicrobial compounds and diminish acquisition and dissemination of antibiotic resistance genes by conjugation.

## Introduction

Bile acids are natural detergents that constitute approximately 50% of the organic components of bile and are involved in digestion and absorption of fats, nutrients, and vitamins. In addition, bile acids serve as signaling molecules that regulate lipid, glucose, energy, metabolic, and inflammatory pathways (Chiang, 2009). Primary bile acids are acidic steroids that are biosynthesized *de novo* in the liver from cholesterol via multiple enzymatic steps and pathways (Monte et al., 2009).

In humans, the two primary 24-carbon bile acids produced are chenodeoxycholic acid and cholic acid. To increase their water solubility under physiological pH conditions, in the final step of their synthesis, cholic acid and chenodeoxycholic acid are conjugated by N-acyl amidation with the hydrophilic amino acids glycine or taurine to form the conjugated bile acids glycocholic and taurocholic acids, or glycochenodeoxycholic and taurochenodeoxycholic acids, respectively (Chiang, 2009).

Secondary bile acids are formed by modification of primary bile acids by intestinal bacteria of conjugated cholic and chenodeoxycholic acids at their 7α-position to form deoxycholic acid, lithocholic acid, and ursodeoxycholic acid (Hofmann and Hagey, 2008). Conjugated bile acids are actively excreted from liver hepatocytes into bile and are concentrated for storage in the gallbladder. After a meal, gallbladder contraction discharges bile acids into the small intestinal lumen; however, when passing through the small intestine, most of the bile acids are reabsorbed by both active and passive mechanisms and return to the liver, where they are resecreted into the bile (Ticho et al., 2019).

Since conjugated bile salts are amphipathic, they can act as surfactants that dissolve lipid particles in water. Thus, the main physiological role of bile acids is to assist in the emulsification of dietary fats and liposoluble vitamins into micelles and help their absorption in the gut (Hofmann and Hagey, 2008). Additionally, the function of bile acids as detergents enables them to interact with the lipid membrane of Gram-positive bacteria and act as potent antimicrobial compounds that prevent the overgrowth of bacterial flora in the small bowel and biliary tree (Begley et al., 2005). In contrast to Gram-positive micro-organisms, less information is known about bile tolerance of Gram-negative bacteria (Begley et al., 2005). Nonetheless, enteric bacteria that colonize the mammalian intestine have evolved to become inherently more resistant to bile than Gram-positive bacteria and can tolerate the antibacterial activities of bile salts at physiological concentrations through various cellular mechanisms (Begley et al., 2005; Urdaneta and Casadesus, 2017; Gipson et al., 2020). Leveraging this difference, bile is often used for selection of enteric Gram-negative bacteria by adding bile salts to bacteriological enrichment media such as MacConkey agar, *Salmonella-Shigella* agar, violet red bile agar and Xylose Lysine Deoxycholate (XLD) Agar (Bridson, 2006).

Previously, we showed that the presence of bile in LB agar medium can reduce the conjugation frequency of the epidemic plasmid pESI from *Salmonella enterica* serovar Infantis from Salmonella enterica serovar Infantis (*S.* Infantis) donor to a recipient *Escherichia coli* strain (Aviv et al., 2016).

(*S*. Infantis) donor to a recipient *Escherichia coli* strain (Aviv et al., 2016). However, the bile component responsible for this phenotype and the effect of bile on the conjugation frequency of other plasmids remained elusive. Here, we aimed to study the ability of different bile components to inhibit acquisition of antibiotic resistance by plasmid conjugation and characterize the effect of active bile components on the conjugative pili, bacterial growth, membrane permeability and antibiotic susceptibility of Gram-negative enteric bacteria.

## Materials and methods

### Bacterial strains, plasmids and culture conditions

The bacterial strains and plasmids used in the present study are listed in **Supplementary Table S1**. Bacterial cultures were routinely maintained in Luria-Bertani (LB) liquid medium or on LB agar plates. For the conjugation experiments, LB agar plates were supplemented with the following antibiotics: ampicillin, 100 µg/ml; kanamycin, 50 µg/ml; tetracycline, 10 µg/ml; rifampicin, 100 µg/ml; and chloramphenicol, 25 µg/ml. To analyze bacterial growth in the presence of glycocholic acid (GCA), overnight cultures that were grown aerobically in LB medium at 37°C were diluted 1:100 in fresh LB and LB supplemented with 2% GCA sodium salt (Merck). Aliquots (200 µL) were dispensed into a 96 well plates that were incubated at 37°C with shaking in an Infinite M Plex (Tecan) plate reader. Optical density (600 nm) was measured every 30 min for up to 25 h. To analyze culture growth on solid LB agar medium, overnight cultures that were grown in LB medium at 37°C were diluted 1:100 into fresh LB, and aliquots of 10 or 30 µL were spotted onto LB agar plates or LB agar supplemented with GCA sodium salt. At designated time points, the bacteria were scraped using inoculation loop, resuspended in 1 ml saline (0.9% NaCl), and serial dilutions were plated onto LB agar plates for CFUs counting.

### Analysis of minimum inhibitory concentration and fractional inhibitory concentration index of antimicrobial compounds

The broth microdilution method was conducted in 96-well microtiter plate format (Wiegand et al., 2008) and was used to analyze the MIC of GCA. Overnight cultures that were grown aerobically in LB medium at 37°C were diluted 1:100 in fresh LB medium and LB supplemented with twofold serial dilutions of GCA sodium salt (Merck) ranged from 0.25 to 16% (w/v). Culture aliquots (200 µl) were dispensed into a 96-well flat-bottom microtitration plates that were incubated at 37°C with shaking in a plate reader. Optical density (600 nm) was measured continually every 30 min for 20 h. MIC was defined as the lowest GCA concentration that completely prevented the growth of the studied bacteria as determined by spectrophotometric measurements. The MIC of ampicillin and chloramphenicol was determined in a similar manner in the presence of twofold serial dilutions of ampicillin, which ranged from 3.125 to 100 µg/ml and chloramphenicol, which ranged from 3.125 to 400 µg/ml. All MIC experiments included at least three biological repeats and the experiments were performed at least twice on different days and with individually prepared inocula.

The FIC index was calculated according to (Meletiadis et al., 2010) and was used to determine the overall interaction between GCA and ampicillin or chloramphenicol and to examine possible synergistic or additive effect in their antimicrobial activities. The FIC of each antibiotic was calculated by dividing the MIC of ampicillin or chloramphenicol in combination with 2% GCA by the MIC of the tested antibiotic alone. FIC index was calculated by adding the calculated FIC of the tested antibiotic together with the calculated FIC of GCA. FIC index ≤ 0.5 indicates synergistic effect while FIC index of 0.5 to 1.0 indicates additive effect.

### Conjugation assay

An agar mating method was conducted as we previously reported (Piscon et al., 2023). Briefly, one ml of donor and recipient cultures grown in LB medium was concentrated, washed, and resuspended in 100 µL of fresh LB. Donor and the recipient cultures were mixed at equal volumes in a test tube, and 20 µL of the conjugation mix or 10 µL of the donor suspension only were spotted onto an LB agar plate or an LB agar plate supplemented with GCA (or other bile components) and incubated at 37°C. After 6 h, the conjugation mix was scraped from the plate and resuspended in 1 ml saline, and serial dilutions were plated onto LB supplemented with the appropriate antibiotics for CFU counting. The plates were incubated at 37°C for overnight, and the conjugation frequency was calculated as the number of transconjugant CFUs/donor CFUs under each condition. Each mating experiment included 3-4 biological repeats, while each experiment was repeated at least twice at different days.

### Motility testing

Bacterial motility on soft agar plates was evaluated as previously reported (Elhadad et al., 2015). Briefly, *E. coli* K1037 was grown in LB under tetracycline selection for overnight at 37°C. The next day, 10 µL of the culture was spotted in the middle of a 0.3% agar LB plate containing different concentrations of GCA (0, 0.2, 1, and 2%). The plates were incubated at 37°C for 16 h, the motility radius was measured, and the plates were imaged.

### Molecular biology and microbial identification

Primers used in this study are listed in **Supplementary Table S2**. Phusion Hot Start Flex DNA polymerase (New England BioLabs) was used for PCR amplification. Environmental isolates were identified using the VITEK mass spectrometry microbial identification system MS ACQ01 (bioMerieux) and 16S rRNA gene sequencing was performed for the sewage isolated bacteria (**Supplementary Table S1**) using the primers 8F_16s_rRNA and 518R_16s_rRNA.

### Quantitative real-time PCR analysis

RNA was extracted from a subculture of *E. coli* K1037 that was grown in LB at 37°C for 3 h. RNA Protect Bacteria Reagent and RNeasy mini kit (Qiagen) were used to extract RNA from 500 µL culture according to the manufacturer’s instructions. An on-column DNase (Qiagen) step and an additional TURBO DNase (Invitrogen) treatment were performed to reduce any genomic DNA contamination. cDNA was synthesized from 200 ng of purified RNA using a qScript cDNA Synthesis Kit (Quanta-bio), according to the manufacturer’s protocol. Real-time PCR was performed on a StepOne Real-Time PCR platform (Applied Biosystems). The results were normalized to the expression of the housekeeping gene, *16S* rRNA. Fold-differences in gene expression compared to the control were calculated as 2^−ΔΔCT^ as previously described (Elhadad et al., 2016).

### 2HA tagging of TraL

A C-terminal 2-hemagglutinin (2HA)-tagged version of TraL was constructed by amplification of *traL* using the primers traL+prom F and traL+prom R, using *E. coli* K1037 harboring pN3 as a DNA template. The insert and the construct pWSK29:2HA tag (Elhadad et al., 2015) were digested with XbaI and SacI (New England BioLabs) for 1 h at 37°C and ligated with T4 DNA Ligase (New England BioLabs).

### Western blotting

An overnight grown culture of *E. coli* K1037/pN3 was subcultured (1:100) in 10 ml fresh LB medium or LB supplemented with GCA. For the supernatant loading control, a spike of 100 mg BSA was added to each culture, and the cultures were grown in a shaker incubator for 8 h at 37°C. At designated time points, 1.1 ml was used for optical density reading (at 600 nm) and for further analyses. One milliliter of the collected sample was centrifuged at 10000 g for 2 min, and the supernatant and pellet were separated. Each pellet was suspended in 200 µL of 1× sodium dodecyl sulfate-polyacrylamide gel electrophoresis (SDS-PAGE) sample buffer [150 mM Tris PH 6.8, 30% glycerol, 1.2% SDS, 2.2M 2-mercaptoethanol, 0.0018% bromophenol blue] and 100 µL of the supernatants were mixed with 100 µL of 2× sample buffer. Protein samples were incubated at 95°C for 10 min and separated on 12% polyacrylamide gel. The gel was stained with Coomassie Blue (Bio-Rad Laboratories), imaged using a Fusion Solo X (Vilber) system, and transferred to a polyvinylidene fluoride (PVDF) membrane (Bio-Rad Laboratories). Immunoblotting was performed using a mouse anti-DnaK antibody (ab69617; Abcam, diluted 1: 5,000) or an anti-HA tag antibody (Abcam; ab18181, diluted 1: 1,000). Conjugated horseradish peroxidase goat anti-mouse antibody (ab6789; Abcam, diluted 1: 10,000) was used as the secondary antibody. Chemiluminescence was detected using enhanced chemiluminescence (ECL) reagents (Bio-Rad Laboratories) and imaged on a Fusion Solo X system. For the gel loading control, membranes were stained with Ponceau S solution (Sigma-Aldrich) and de-stained with dH_2_0.

### NPN and Ethidium Bromide uptake assays

An overnight grown culture of *E. coli* K1037 was subcultured into fresh LB or LB supplemented with 2% GCA and grown to an OD_600_ of ~1. The cultures were harvested by centrifugation, washed twice with an assay buffer (5 mM HEPES, 5 mM glucose, pH 7.2), and resuspended in 1 ml assay buffer. Aliquots (200 µL) were transferred to a black 96 well microplate (Greiner), and 1 µL of 2 µM N-Phenyl-1-naphthylamine (NPN; Sigma-Aldrich) was added to each well to a final concentration of 10 nM. The fluorescence intensity was immediately read using an Infinite M Plex (Tecan) plate reader at an excitation wavelength of 350 nm and an emission wavelength of 420 nm. The fluorescence intensity at each time point was normalized to the bacterial optical density, measured at 600 nm.

For EtBr uptake assay, the cultures were washed with PBS and resuspended in PBS containing increasing concentrations of glycocholic acid. EtBr was then added at a final concentration of 2 µg/ml. Fluorescence was measured at excitation and emission wavelengths of 520 and 600 nm, respectively. The fluorescence intensity at each time point was normalized to the bacterial density that was measured at 600 nm.

### Electron microscopy

*E. coli* K1037 harboring N3 plasmid was grown in LB medium at 37°C for overnight. For analysis in liquid cultures, the bacteria were diluted 1:100 in fresh LB or into LB supplemented with 2% GCA and incubated at 37°C for 6 h. 100 µL from the liquid subculture was added to 250 µL phosphate-buffered saline (PBS). For analysis of LB agar grown cultures, 30 µL of the subculture was spotted onto an LB agar plate or LB agar plate supplemented with 2% GCA, incubated at 37°C for 6 h, and then very gently scraped into 250 µL PBS. The bacterial suspension (100 µL) was applied onto fibronectin-coated 13 mm round “Deckglaser” coverslips, incubated for 1 h at room temperature, and fixed in a fixation buffer for 20 min at room temperature. Samples were then washed with 1 ml of 0.1 M sodium cacodylate buffer pH 7.4), followed by a secondary fixation with 2% osmium tetroxide in 0.1 M sodium cacodylate for 10 min. The samples were washed twice with double-distilled water and dehydrated under a series of increasing ethanol concentrations (ranging from 7.5% to 100%). The samples were then subjected to critical-point drying using a K850 critical-point dryer (Quorum Technologies) and coated with ~1 nm iridium using a Q150T coater (Quorum Technologies). The samples were imaged using a *Merlin* scanning electron microscope (Zeiss). For reliable quantification, we counted only “functional” conjugative pili that were visually confirmed to connect two bacterial cells, and did not consider broken or disconnected pili. To facilitate quantitative, unbiased analysis, large fields of bacteria were scanned at a magnification of 10,000× with a scan resolution of 12,288 × 9,216 pixels. These settings enabled zooming in and out and digitally magnifying the images to an equivalent of 100,000×, revealing details of the conjugative pili and membrane texture. Thus, large numbers of bacteria could be systematically analyzed and scored individually for the occurrence of pili or the punctured phenotype.

### Isolation of bacteria from the sewage system

Kitchen cellulose sponges (Scotch-Brite) with a size of 7 × 5.5 cm were soaked with saline (control) or saline supplemented with 1% GCA (experiment). Six sponges from each treatment were placed and stabilized approximately 10 cm above the bottom of five different sewage pits in the central sewage pipeline of Sheba Medical Center. After 20 or 39 days, the sponges were removed and vigorously washed with 50 ml saline for 60 s to extract the occupying bacteria. The extracts were centrifuged at 16000 g for 15 min at 4°C, washed with saline, and resuspended in a final volume of 1 ml saline. The concentrated samples were plated on MacConckey agar plates (BD) that were incubated at 37°C for 16 h. Obtained colonies were picked up and replica plated on CHROMagar plates (PD165, Hylabs) and MacConckey agar plates under chloramphenicol selection (25 µg/ml). The number of chloramphenicol-resistant colonies obtained from saline-and GCA-immersed sponges was determined.

## Results

### GCA inhibits Enterobacteriaceae conjugation

**Figure 1:**
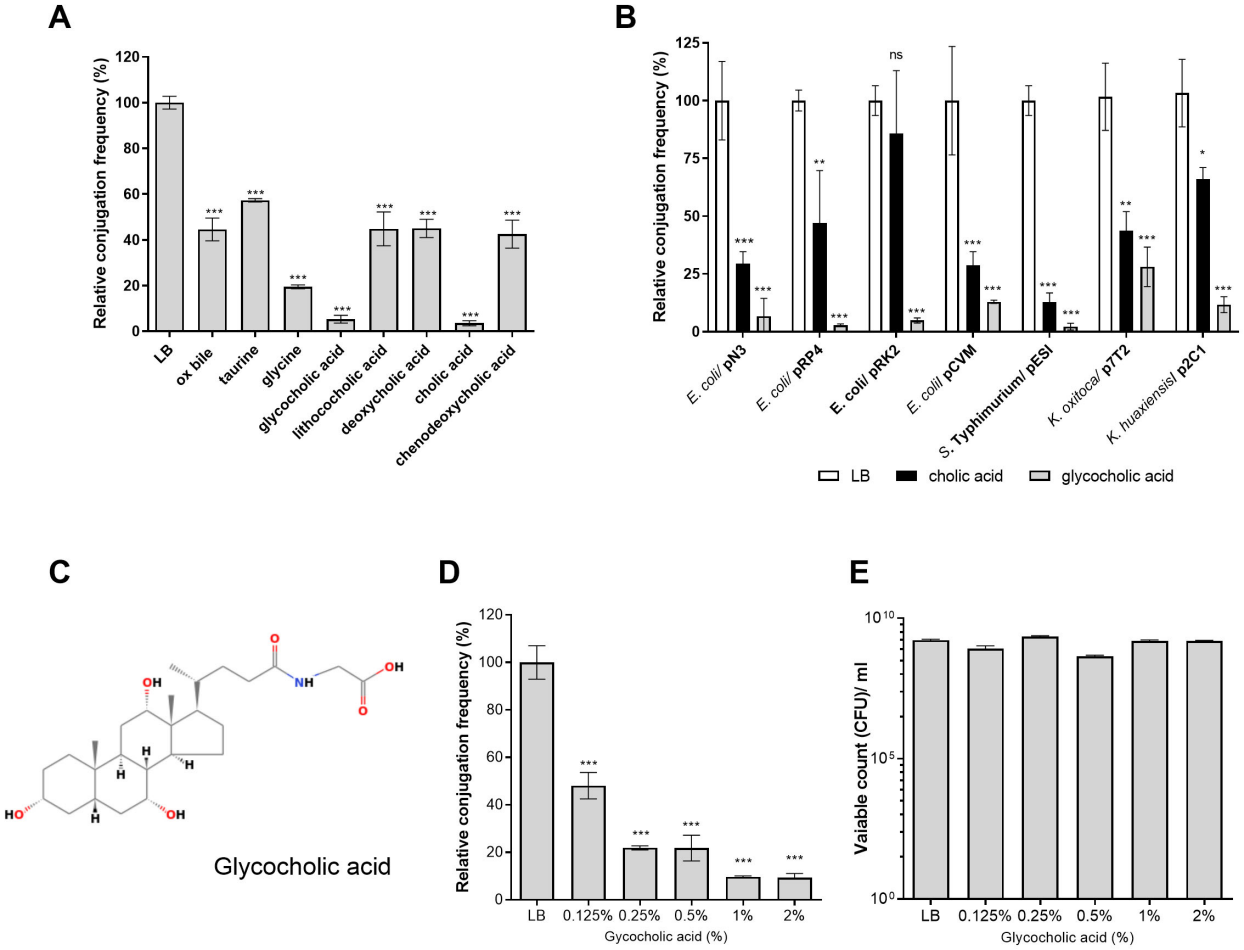
GCA inhibits bacterial conjugation. **(A)** Plate mating assays between *E. coli* K1037 harboring pN3 (Tc^R^; donor) and *E. coli* K-12 ORN 172 (Km^R^; recipient) were conducted on LB agar plates supplemented with 2% (W/V) bile or bile components at 37°C for 6 h. Conjugation frequency was calculated as the number of obtained transconjugants/number of donor CFUs and is shown relative to the conjugation frequency on LB-agar, which was normalized to 100%. The graph shows the mean and the standard error of the mean (SEM) of at least three independent biological experiments. One-way ANOVA against the conjugation frequency on LB was used to determined statistical significance. ***p <0.001. **(B)** Transfer frequency of various conjugative plasmids were studied with different donor strains including *E. coli* K1037/pN3, *E. coli* FS1290/pRP4, *E. coli* DH10B/pCVM29188, *Salmonella* Typhimurium SL1344/pESI, *Klebsiella oxytoca* SMCSS-7T2/p7T2 and *Klebsiella huaxiensis* SMCSS-2C1/p2C1 and the recipient strains *E. coli* K-12 ORN 172 (Km^R^) or *E. coli* J-53 (Rif^R^). The mating strains and each one of the donors alone, were spotted onto LB agar plates (white bars), LB agar plates supplemented with 2% cholic acid (black bars) or LB agar plates supplemented with 2% GCA (grey bars) and conjugation was conducted as above. The chart shows the mean and SEM of three independent biological repeats. One-way ANOVA was used to determined statistical significance against the conjugation frequency of each mating on LB plate that was normalized to 100%. ns, not significant; *p<0.05; **p<0.01; ***p<0.001. **(C)** The chemical structure of GCA (ChemSpider ID 9734) is shown. **(D)** Plate mating was performed on LB agar supplemented with increasing concentrations of GCA sodium salt (0, 0.125, 0.25, 0.5, 1 and 2%) as in **(A)**. The chart shows the mean and SEM of at least three independent biological repeats. One-way ANOVA was used to determined statistical significance. ***p<0.001. **(E)** CFUs count of the donor strain (*E. coli* K1037 harboring pN3) following 6 h incubation on LB agar plates with different concentrations of GCA sodium salt that was done in parallel to the mating experiment shown in **(D)**. The graph shows the mean and SEM of 3-4 independent biological repeats.

Previously, we showed that the presence of bile in LB agar medium can reduce the conjugation frequency of the epidemic plasmid pESI from the *S*. Infantis donor to a recipient *E. coli* strain (Aviv et al., 2016). This observation led us to study the generality of this phenomenon and identify bile compounds that inhibit conjugation. Therefore, we tested the effect of a complete ox bile and seven bile components (cholic acid, lithocholic acid, deoxycholic acid, chenodeoxycholic acid, glycocholic acid, taurine, and glycine) on the conjugation frequency of the broad host-range IncN plasmid, pN3, which we have recently characterized (Piscon et al., 2023). Since the conjugation frequency of pN3 was found to be much higher on solid media than in liquid (Piscon et al., 2023) all downstream conjugation analyses were performed on 1.5% agar LB plates. As we have previously shown for pESI (Aviv et al., 2016), we found that pN3 conjugation was significantly inhibited in the presence of 2% ox bile and its other tested components at varying levels. Nevertheless, the most prominent inhibitory effect on pN3 conjugation was observed in the presence of 2% cholic acid or GCA (as sodium salts), which reduced its conjugation by approximately 95% (**Figure 1A**; **Supplementary Figure S1**).

To further examine the universality of this finding, we have determined the conjugation frequency of seven conjugative plasmids from at least five different incompatibility (Inc) groups (**Supplementary Table S1**) harbored by various Enterobacteriaceae bile-resistant species (*Escherichia coli*, *Salmonella enterica*, *Klebsiella oxytoca* and *Klebsiella huaxiensis*) in the presence of 2% cholic or glycocholic acids (**Figure 1B**). These experiments indicated that glycocholic acid, sodium salt (**Figure 1C**) was the most potent conjugation inhibitor that reduced the conjugation frequency of all the tested plasmids by 70 to 97% in comparison to their conjugation frequency in the absence of these bile acids. In addition, different experiments demonstrated more consisting inhibitory effect (and smaller standard deviation) with GCA than cholic acid and therefore we decided to further characterize the effect of GCA on pN3 conjugation.

Follow up experiments using pN3 as a model plasmid have demonstrated that increasing the concentrations of GCA in the LB agar conjugation plates from 0 to 2% resulted in a dose-dependent reduction in the conjugation frequency (**Figure 1D**). Additionally, we confirmed that the presence of these GCA concentrations in LB agar plates was not bactericidal, as they did not cause a prominent reduction in *E. coli* K1037 viability, which was used as the donor strain (**Figure 1E**).

To test if the conjugation inhibition is due to the amphipathic property of glycocholic acid, pN3 conjugation was also tested in the presence of the non-ionic surfactant Triton X-100 (C_16_H_26_O_2_). In contrast to glycocholic acid, 0.5-2% Triton X-100 did not inhibit the conjugation frequency of pN3 (**Supplementary Figure S2**), indicating that the conjugation inhibition by GCA is not conferred merely by its detergent activity and that additional physicochemical properties are likely involved. Together, we concluded from these results that the presence of GCA in solid media potently inhibits bacterial conjugation of various conjugative plasmids without affecting the viability of these strains.

### GCA represses bacterial motility and *fliC*, but not *tra* genes expression

**Figure 2:**
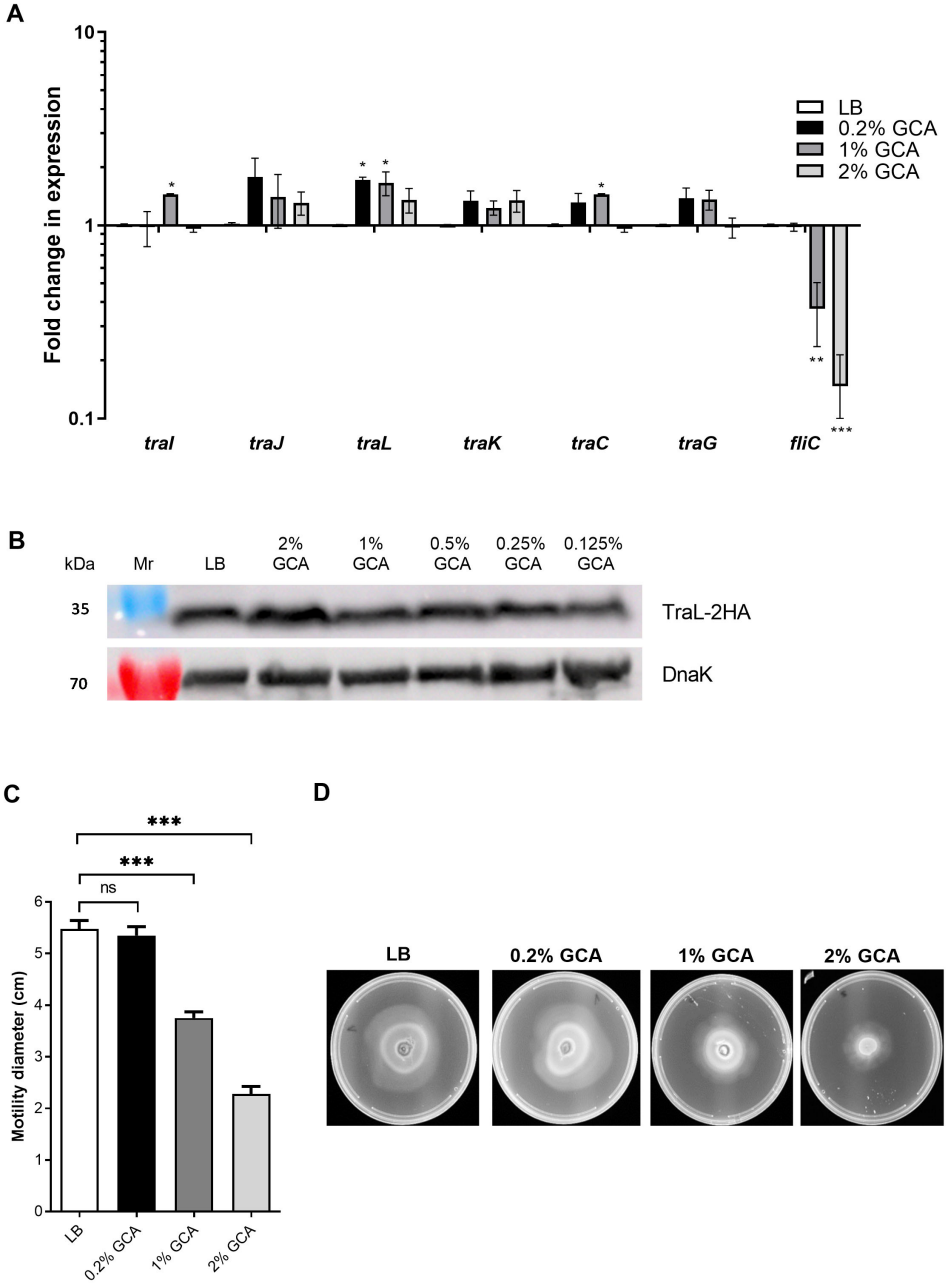
GCA inhibits motility and *fliC*, but not *tra* genes expression. **(A)** RNA was extracted from *E. coli* K1037 harboring pN3 cultures that were grown to the late logarithmic phase in LB medium supplemented with varying concentrations of GCA. The expression of six *tra* genes (*traI, traJ, traL, traK, traG* and *traC*) encoded in pN3 and the chromosomally encoded flagellin, *fliC* gene was measured using qRT-PCR and normalized to the expression of the 16S rRNA housekeeping gene. The chart shows the mean and SEM of three biological repeats in one representative experiment out of three. One-way ANOVA against the expression in LB was used to determined statistical significance. Unsigned bars indicate not significant change in expression; *p<0.05; **p<0.01; ***p<0.001. **(B)** *E. coli* K1037 expressing C-terminus 2HA tagged version of TraL were subcultured in LB broth with interchanging GCA concentrations and grown for 3 h at 37°C. The cultures were OD (600 nm) normalized and analyzed by Western blotting using anti-HA antibody or anti-DnaK antibody that was used as a loading control. **(C)** Motility of *E. coli* K1037 was examined on soft agar (0.3%) LB plates supplemented with increasing concentrations of GCA after 16 h incubation at 37°C. The mean swimming diameter (in cm) and the SEM of three independent plates per condition is shown. One-way ANOVA was used to determined statistical significance. ns, not significant; ***p<0. 001. **(D)** A representative image of the swimming on soft LB agar plates is shown for each tested condition.

Previous studies have shown that bile or GCA represses the expression of flagellin genes *fljB*:z66 and *fliC* in enterotoxigenic *E. coli* (Joffre et al., 2019), enterohemorrhagic *E. coli* O157:H7 (Hamner et al., 2013) and *S*. Typhi (Xu et al., 2008), indicating that bile or its constituent compounds can alter gene expression in Enterobacteriaceae species. Therefore, we investigated whether the reduced conjugation frequency was the result of conjugation gene (*tra*) repression in response to GCA. Quantitative real-time reverse transcription PCR (qRT-PCR) was used to determine the expression of six conjugation genes (*traI, traJ, traL, traK, traC*, and *traG*) in *E. coli* K1037 harboring pN3 in the presence of 0, 0.2, 1 and 2% GCA. In addition, the flagellin gene, *fliC* was included as a positive control. As shown in **Figure 2A**, while the expression of *fliC* was significantly downregulated in the presence of 1 and 2% GCA, no biologically-significant changes (more than 2-fold) in the expression of the tested *tra* genes were observed.

To further examine the possible effect of GCA on the expression of *tra* genes post-transcriptionally, we tagged the TraL protein of pN3 with an influenza hemagglutinin (2HA) tag. TraL is a putative lytic transglycosylase and an *Agrobacterium tumefaciens* VirB1 ortholog, which was previously shown to be required for the assembly of the conjugative type-four secretion system (Hoppner et al., 2004). Western blotting analysis using an anti-2HA tag against protein extracts from *E. coli* K1037 grown in the presence of varying concentrations of GCA indicated a constitutive expression of TraL-2HA that did not change in the presence of GCA (**Figure 2B**).

In contrast to the stable expression of *tra* genes, the significant reduction in *fliC* expression in the presence of GCA led us to examine the motility of these bacteria on semi-solid agar plates in the presence of GCA. Notably, in agreement with the reduced expression of *fliC*, we observed significant reduction in bacterial motility when GCA was added at a final concentration of 1 or 2% (**Figures 2C, D**). We concluded from these experiments that while GCA downregulated the expression of *fliC* and reduced the motility of *E. coli*, it did not change the expression of *tra* genes directly. Therefore, a different mechanism is likely responsible for the reduced bacterial conjugation in the presence of GCA.

### GCA reduces the occurrence of the conjugative pili

**Figure 3:**
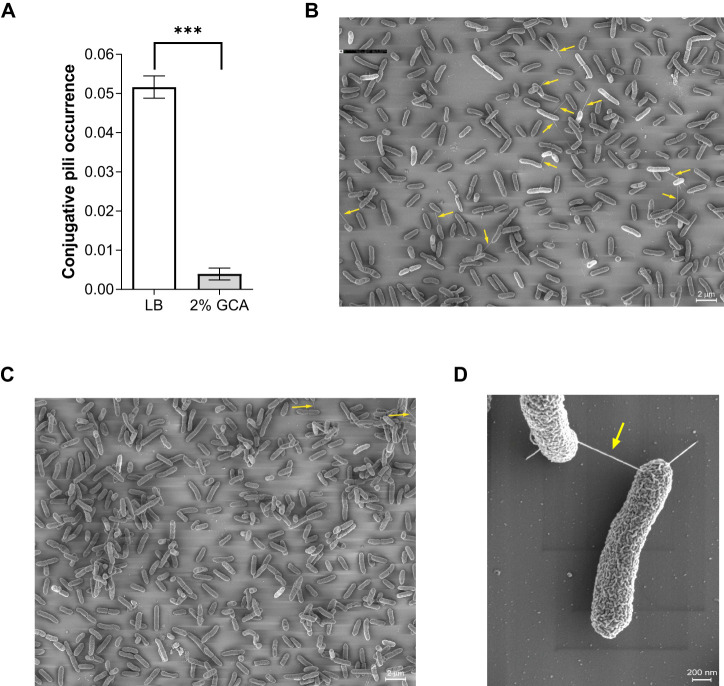
GCA reduces the occurrence of the conjugative pili. Overnight culture of *E. coli* K1037 harboring pN3 plasmid was diluted 1:100 and spotted (30 µL) onto an LB agar plate and LB agar supplemented with 2% GCA sodium salt that were incubated at 37°C for 6 h. The samples were fixed on fibronectin-coated coverslips, sputter coated with iridium and imaged using a *Merlin* scanning electron microscope. **(A)** The number of conjugative pili among 4237 imaged individual bacteria that were incubated on LB agar plates and among 3249 imaged bacteria that were incubated on LB agar plates containing 2% GCA was counted and is shown as the mean ratio between the number of conjugative pili and the total number of counted cells in each field. The bars show the mean and SEM in ten independent SEM fields. Unpaired t-test was used to determined statistical significance. ***p<0.001. **(B)** A representative scanning EM field of the bacteria following incubation on an LB agar plate. Conjugative pili are indicated by a yellow arrow. **(C)** A representative scanning EM field of bacteria following incubation on an LB agar plate supplemented with 2% glycocholic acid. Conjugative pili are indicated by a yellow arrow. **(D)** Enlarged image of a pN3-mediated conjugation pili connecting two mating bacteria.

Recently, we demonstrated the formation of conjugative pili mediated by pN3 (Piscon et al., 2023). Here, we applied a similar approach and used thin iridium coating and high-resolution scanning electron microscopy (scanning EM), to image and quantify the formation of pN3 conjugation pili in the absence or presence of GCA (2%) on solid mating agar plates. Overall, conjugative pili were evaluated in ten independent scanning EM fields, consisting in total 4237 individual imaged bacteria from cultures that were incubated on LB agar plates and 3249 imaged bacterial cells from cultures that were incubated on LB agar plates containing 2% glycocholic acid. Strikingly, we observed that while the occurrence of conjugative pili was 0.05 (pili/total number of bacteria) in cultures incubated on LB plates, their occurrence was only 0.003 in the presence of glycocholic acid, demonstrating a more than 10-fold reduction in the frequency of conjugative pili (**Figure 3**). These results suggested that the decrease in conjugation frequency may be the result of poorer conjugative pili formation or integrity in bacteria grown in the presence of glycocholic acid.

### GCA inhibits gram-negative bacteria growth in liquid, but not on solid media

**Figure 4:**
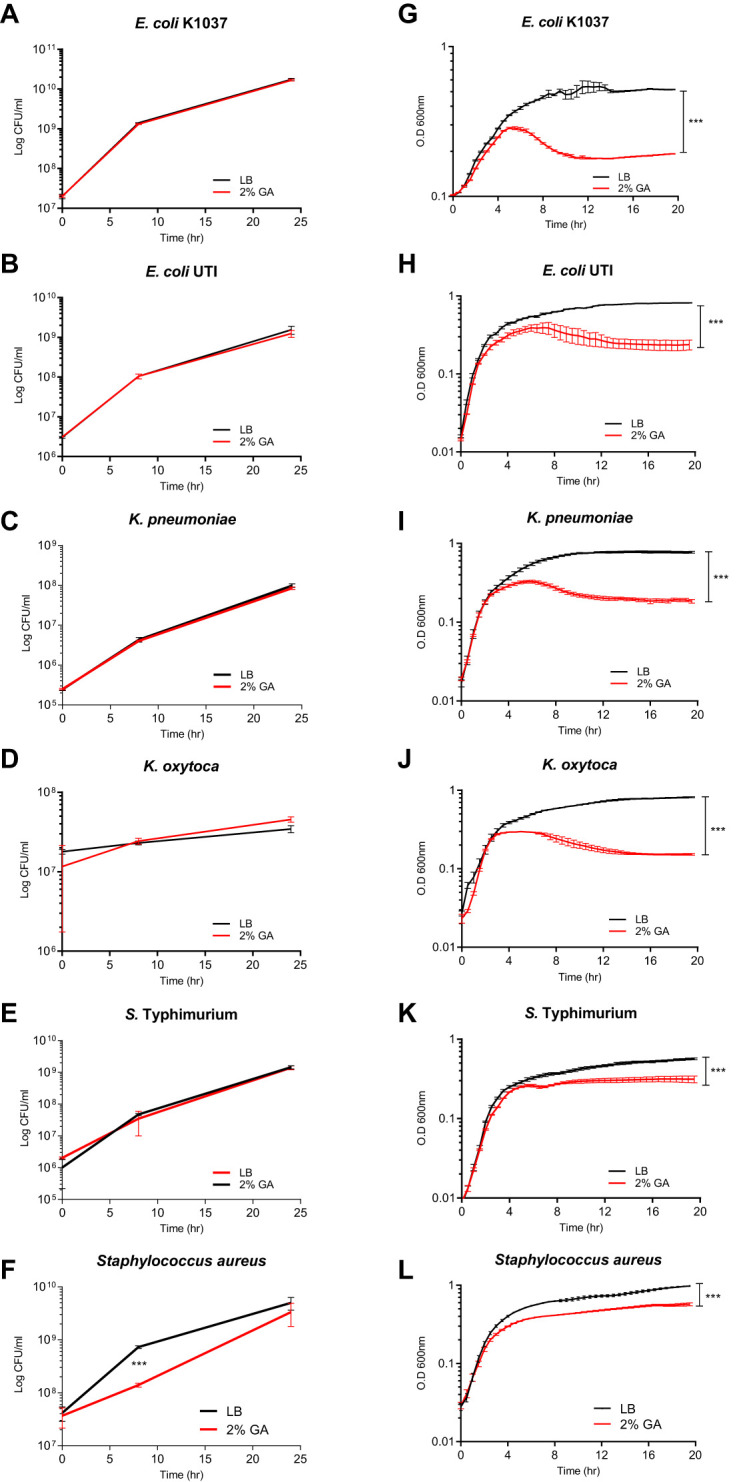
GCA inhibits Enterobacteriaceae growth in liquid culture but not on solid medium. *E. Coli* K1037 **(A)**, a clinical UTI *E. coli* isolate **(B)**, *Klebsiella pneumonia* ATCC700721 **(C)**, *Klebsiella oxytoca* SMCSS-7T2 **(D)**, *Salmonella* Typhimurium SL1344 **(E)** and the Gram-positive bacterium, *Staphylococcus aureus* ATCC 25923 that was included as a positive control **(F)**, were cultured at 37°C in LB medium and spotted (30 µL) onto solid LB agar plates (black lines) or LB agar supplemented with 2% GCA (red lines). At time points 0, 8, and 24 h post plating, the bacteria were scraped from the plates, resuspended in 1 ml saline, serially diluted and plated onto LB plates for CFUs counting. The charts present the mean and SEM of two biological repeats. **(G–L)** The same strains as above were subcultured and grown at 37°C in liquid LB medium (black curves) or liquid LB supplemented with 2% GCA (red curves). Optical density at 600 nm was measured every 30 minutes for 20 h. The charts present the mean and SEM of six repeats. All experiments were conducted at least two times. Two-way ANOVA was used to determined statistical significance. ***p<0.001.

To better understand the effect of GCA on the physiology of Enterobacteriaceae species, we characterized the growth of *E. coli* K1037, a clinical isolate of *E. coli* from a urinary tract infection (UTI) patient, *Klebsiella pneumonia*, *Klebsiella oxytoca*, and *S. enterica* serovar Typhimurium (*S*. Typhimurium) in the absence and presence of 2% GCA sodium salt in liquid and on solid LB agar medium. Interestingly, although the growth of these strains was not inhibited on solid LB agar medium containing GCA (**Figures 4A–E**), their growth was significantly inhibited in liquid culture supplemented with 2% GCA (**Figures 4G–K**). Specifically, growth inhibition in all tested strains was observed during the late logarithmic phase, before entering into the stationary stage after ~ 4-6 h of subculturing. A control experiment that was conducted with the Gram-positive bacterium, *Staphylococcus aureus* showed growth inhibition both on LB agar plates and in liquid LB medium (**Figures 4F, L**). We concluded from these experiments that, while Enterobacteriaceae are considered relatively bile-resistant and can readily grow on agar plates supplemented with glycocholic acid, in liquid medium, the growth of many Enterobacteriaceae species is significantly inhibited at their late logarithmic phase.

### GCA increases membrane permeability of gram-negative bacteria

**Figure 5:**
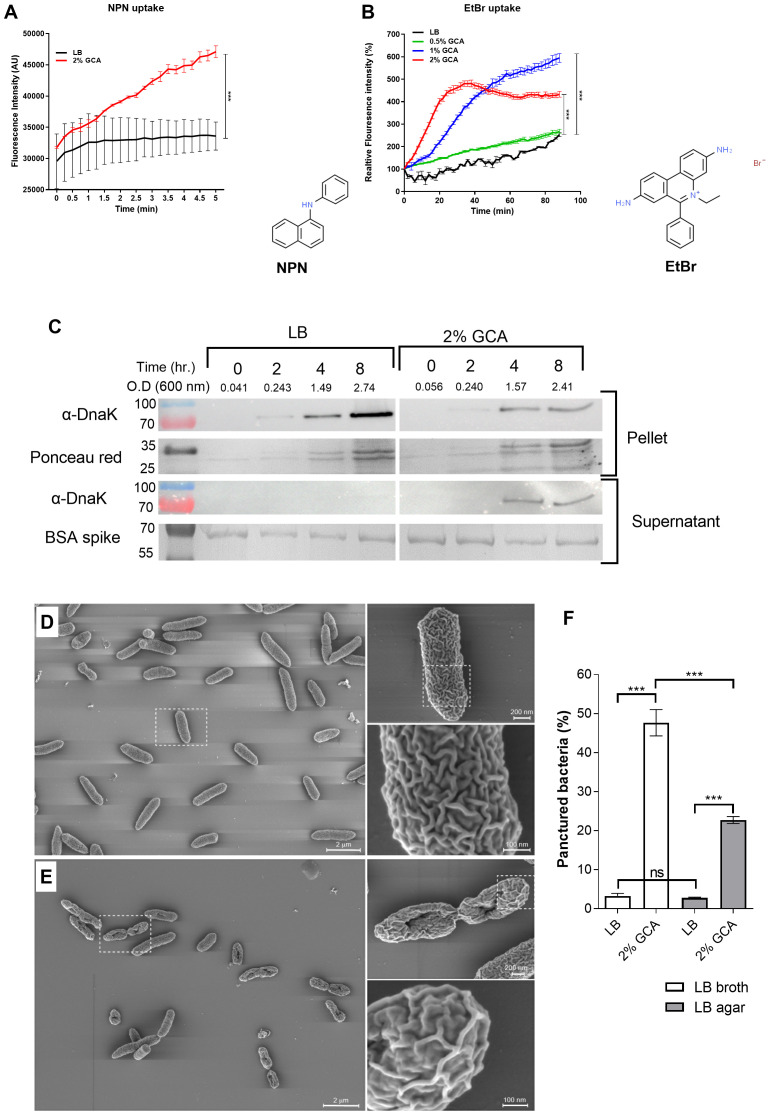
GCA increases Enterobacteriaceae membrane permeability and changes its morphology. **(A)** *E. coli* K1037 cultures that were grown in LB (black curve) or in LB supplemented with 2% GCA (red curve) for 16 h at 37°C were washed and N-Phenyl-1-naphthalenamine (NPN) was added to the samples to a final concentration of 10 nM. Fluorescence intensity (excitation 350 nm, emission 420 nm) and optical density (600 nm) were measured every 25 seconds. The Fluorescence intensity at each time point was normalized to the optical density at 600 nm and is shown as arbitrary units (AU). The graph presents the mean and SEM of three independent biological repeats. Two-way ANOVA was used to determined statistical significance ***p < 0.001. The chemical structure of NPN (ChemSpider ID 6746) is shown to the right of the graph. **(B)** *E. coli* K1037 culture that was grown in liquid LB for 16 h at 37°C were washed and resuspended with PBS (black line), or in in PBS supplemented with 0.5% (green line), 1% (blue line) or 2% (red line) GCA in the presence of 20 µM ethidium bromide (EtBr). The fluorescence intensity (excitation 520 nm, emission 600 nm) and optical density (at 600 nm) of the samples was measured every two minutes and is presented relative to T0. The chart presents the mean and SEM of three biological repeats. Two-way ANOVA was used to determined statistical significance. ***p < 0.001. The chemical structure of EtBr (ChemSpider ID14034) is shown to the right of the graph. **(C)** *E. coli* K1037 that was grown in liquid LB for overnight at 37°C was subcultured into fresh liquid LB or LB supplemented with 2% GCA and incubated at 37°C. As a loading control for the supernatant fraction, a BSA spike (100 mg) was added to each culture. At designated time points, optical density at 600 nm was measured and aliquots were removed and separated on a 12% SDS-PAGE gel. Western blotting was conducted using α-DnaK antibody probing the cellular (pellet) and the supernatant fractions. As a loading control of the pellet fractions, the membrane was stained with Ponceau red. The BSA loading control of the supernatant is shown following Coomassie blue gel staining. The molecular weight of protein markers (in kDa) are shown to the left of the blots. **(D)** A stationary phase culture of *E. coli* K1037 was subcultured into fresh LB broth or LB broth supplemented with 2% glycocholic acid. **(E)** Similarly, aliquots (30 µL) were spotted onto LB agar and LB agar supplemented with 2% glycocholic acid. All liquid and agar grown cultures were incubated at 37°C for 6 h and imaged by scanning EM after sputter iridium coating. **(F)** The percentage of damaged bacteria that presented a punctured phenotype was counted in ten independent fields of cultures that were grown in liquid LB with (N=1164) and without (N=1727) glycocholic acid, or on LB agar plates that were grown in the presence (N=3249) and absence (N=4237) of glycocholic acid. Unpaired t-test was used to determined statistical significance. ns, not significant; ***p<0.001.

A possible mechanism that could explain the growth inhibition of GCA in aqueous conditions, but not in solid medium, is a potential change in membrane permeability due to the amphipathic nature of bile acids (Hofmann and Hagey, 2008). To test this hypothesis, we used an N-phenyl-1-naphthylamine (NPN) uptake assay. NPN is a nonpolar, hydrophobic fluorescent molecule that has weak fluorescence in aqueous environments, but becomes strongly fluorescent in nonpolar environments, such as membrane lipids (Brito and Vaz, 1986). The increase in NPN fluorescence intensity was studied in *E. coli* K1037 cultures that were incubated in the presence or absence of 2% glycocholic acid, and then washed. Subsequently, a significantly higher NPN fluorescence intensity was measured in cultures exposed to GCA in LB than in cultures grown in liquid LB without GCA (**Figure 5A**).

Similar results were obtained using an Ethidium Bromide (EtBr) uptake assay. EtBr is a DNA intercalator that emits a weak fluorescence in aqueous solution outside the cell but becomes highly fluorescent when crossing the membrane and binds to DNA. Thus, fluorescence intensity is correlated with the transport of EtBr across the cell envelope and is dependent on membrane permeability (Paixao et al., 2009). Conducting this assay with *E. coli* K1037 demonstrated higher fluorescence intensity of EtBr with the elevated concentrations of GCA (**Figure 5B**). A control experiment, in which EtBr was incubated in PBS in the presence of 1 µg *E. coli* DNA and increasing concentrations of GCA (0, 0.5, 1 and 2%) showed no significant change in the florescence intensity (**Supplementary Figure S3**), and together these results suggested that GCA increases the membrane permeability of *E. coli* cells.

To further test possible changes in membrane permeability in the presence of glycocholic acid, we determined using Western blotting the amount of DnaK in the supernatant of bacterial cultures grown in the presence and absence of glycocholic acid. DnaK is a cytoplasmic chaperone that can be dispensed from the cell when the integrity of the bacterial membrane is compromised (Rinas and Hoffmann, 2004). To account for DnaK leakage, *E. coli* K1037 cultures grown in LB with and without GCA were probed with anti-DnaK antibodies for the presence of the DnaK in the cells pellet and supernatant. As shown in **Figure 5C**, while intracellular DnaK was identified in the pellet (cellular fraction) of both cultures, leaked DnaK to the supernatant was detected only in the culture grown in the presence of glycocholic acid, concurring with the possibility that membrane integrity is compromised in the presence of glycocholic acid.

Next, we applied sputter iridium coating, which allows extremely thin, fine-grained, uniform coating of the bacterial membrane, and high resolution scanning EM to characterize the bacterial morphology and membrane texture of *E. coli* cells that were grown in the presence and absence of 2% glycocholic acid. Markedly, many of the cells that were grown for 6 h in the presence of 2% GCA presented a damaged morphology with a single puncture in the center of the cell (**Figure 5**; **Supplementary Figure S4**) and changes in the membrane texture that seemed less “folded” than those of cells grown without GCA (compare the enlarged image in **Figure 5D** with **Figure 5E**). To quantify the damaged morphology, the number of punctured cells was blindly counted in 10 independent microscopy fields of cultures grown in the presence and absence of GCA in liquid (LB) and solid (LB agar) media. This analysis indicated that while only 3% (53/1727) of the cells that were not exposed to GCA in liquid media exhibited an injured morphology, 47% (543/1164) of the cells that were subcultured in the presence of GCA were observed with a puncture in their membrane. Interestingly, as shown in **Figure 5F** and in agreement with the growth curves presented in **Figure 4**, much less damaged cells (23%; 740/3249) were counted in cultures that were exposed to GCA on a solid LB agar medium, in comparison to bacteria that were grown in the presence of GCA in liquid LB. The percentage of punctured cells in cultures grown on LB agar plates without GCA was similar to the one counted in LB liquid cultures and reached 2.8% (119/4237).

Accumulatively these results provide several independent pieces of evidence that support the notion that GCA changes the membrane architecture of Gram-negative bacteria, predominantly in aquatic conditions, in a way that increases its permeability and/or compromises membrane integrity.

### GCA augments the susceptibility of Enterobacteriaceae species to antibiotics

**Figure 6:**
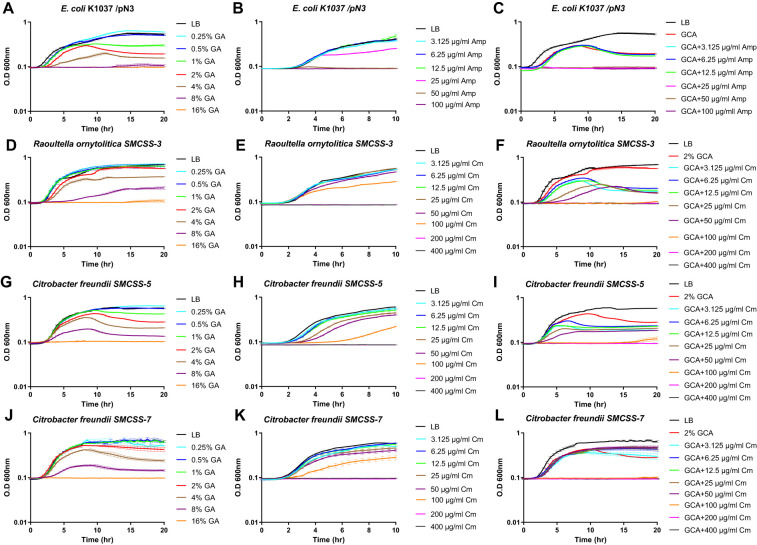
GCA enhances the bacterial susceptibility to antibiotics. *E. coli* K1037 **(A–C)**, *Raoultella ornithinolytica* SMCSS-3 **(D–F)**, *Citrobacter freundii* SMCSS-5 **(G–I)** and *Citrobacter freundii* SMCSS-5 **(J–L)** were grown in LB medium at 37°C in the presence of twofold serial dilutions of GCA ranged from 0.25-16% **(A, D, G, J)**; ampicillin (ranged from 3.125-100 µg/ml) **(B)**; chloramphenicol [ranged from 3.125-400 µg/ml; **(E, H, K)**]; or in the presence of 2% GCA and antibiotics **(C, F, I, L)**. Cultures growth is shown as the change in the optical density at 600 nm over thime that was measured every 30 min. Each time point indicates the mean and SEM of three repeats. The charts show one represented experiment out of 2-3 independent experiments.

Based on the identified unexpected effect of GCA on the Gram-negative bacterial membrane, we also investigated whether GCA can alter the tolerance of resistant bacteria to antimicrobial compounds. We used *E. coli* K1037, which is resistant to low concentrations (up to 25 µg/ml) of ampicillin, and *Raoultella ornithinolytica* and *Citrobacter freundii* strains, which were isolated from the sewage of the Sheba Medical Center and were found to be resistant to up to 200 µg/ml chloramphenicol. The minimum inhibitory concentration (MIC) of GCA and both antibiotics was determined for these strains by the broth microdilution method and their fractional inhibitory concentration (FIC) index was calculated accordingly. In agreement with the results shown in **Figure 4**, the addition of GCA impaired their growth in a dose-dependent manner and caused a growth inhibition at the late logarithmic stage before cultures entered the stationary phase (**Figures 6A, D, G, J**). The addition of ampicillin to *E. coli* K1037 cultures in concentrations above 50 µg/ml, or chloramphenicol to the *R. ornithinolytica* and *C. freundii* cultures in concentrations above 200 µg/ml completely ceased their growth (**Figures 6B, E, H, K**). Nevertheless, the addition of both GCA to a final concentration of 2% and antibiotics reduced the observed MIC of the antibiotics by at least two-fold, indicating additive interaction between GCA and antibiotics in these strains (**Figures 6C, F, I, L**, **Table 1**). These results indicate that GCA can augment the antimicrobial activity of different antibiotics, even in resistant bacteria, most likely due to its effect on the bacterial membrane and the increase in its permeability, which probably elevates antibiotic influx into the cell.

**Table 1:** GCA augments the susceptibility of Enterobacteriaceae species to antibiotics.

Bacterial species and strain	MIC GCA	MIC antibiotic	MIC antibiotic + 2% GCA	FIC antibiotic	FIC GCA	FIC index
*E. coli* K1037	8%	50 µg/ml (Amp)	25 µg/ml (Amp)	0.5	0.25	0.75
*Raoultella ornithinolytica* SMCSS-3	16%	200 µg/ml (Cm)	100 µg/ml (Cm)	0.5	0.125	0.625
*Citrobacter freundii* SMCSS-5	16%	200 µg/ml (Cm)	100 µg/ml (Cm)	0.5	0.125	0.625
*Citrobacter freundii* SMCSS-7	16%	200 µg/ml (Cm)	100 µg/ml (Cm)	0.5	0.125	0.625

*E. coli* K1037, *R. ornithinolytica* SMCSS-3, *C. freundii* SMCSS-5 and *C. freundii* SMCSS-7 were grown in LB medium at 37°C in the presence of twofold serial dilutions of GCA (ranged from 0.25- 16%), ampicillin (ranged from 3.125-100 µg/ml) and chloramphenicol (ranged from 3.125-400 µg/ml). Culture growth was determined by spectrophotometric reading at 600 nm. The MIC of each compound was determined as the minimal concentration that completely prevented the growth of the cultures, while the FIC and the FIC index were calculated as explained in the Materials and Methods section. FIC index between 0.5 and 1 indicates additive effect of GCA and the studied antibiotic.

## Discussion

Bile and bile salts are long known as potent antimicrobial agents against Gram-positive bacteria that play an important role in the innate defense of the intestine (Sung et al., 1993; Begley et al., 2005; Li et al., 2024). The antimicrobial property of bile is widely exploited as a selective inhibitory agent in different bacteriological media such as MacConkey, *Salmonella-Shigella* (SS), and Xylose Lysine Deoxycholate (XLD) agar, which inhibit the growth of Gram-positive bacteria but allow the growth of Gram-negative enteric bacteria, which are considered more resistant to bile relative to Gram-positive bacteria (Begley et al., 2005; Urdaneta and Casadesus, 2017). For instance, Van Velkinburgh and Gunn have shown that the minimal inhibitory concentrations of ox bile for *S*. Typhimurium and *S*. Typhi were 18% and 12%, respectively, and that the minimal bactericidal concentrations (MBCs) were greater than 60% for *S*. Typhimurium and 18% for *S*. Typhi (Van Velkinburgh and Gunn, 1999). These MBCs are much higher than the physiological concentrations of bile in the small intestine that was shown to range between 0.2% and 2% (wt/vol) (Van Deest et al., 1968; Dawson, 1998; Gipson et al., 2020)*. E. coli* is also considered highly resistant to bile (Doranga and Conway, 2023) and is frequently isolated from the gallbladder and bile of animals and humans (Brook, 1989; Cable et al., 1997; Flores et al., 2003). This selective advantage for Gram-negative bacteria is believed to be largely due to their lipopolysaccharide (LPS) outer membrane, which functions as an efficient barrier, and broad-specificity efflux pumps that capture and eject toxic molecules such as bile, infiltrating through the outer membrane (Provenzano et al., 2000; Chatterjee et al., 2004).

Indeed, previous studies have shown that various Gram-negative species, including *E. coli* and *V. cholerae* are resistant to bile acids when they were plated on LB agar plates supplemented with cholate or deoxycholate, or when they were grown in liquid LB in the presence of these bile salts for a relatively short time (until culture reached late log phase) (Provenzano et al., 2000; Cremers et al., 2014). Here, we showed that various Enterobacteriaceae species, including *E. coli, K. pneumonia, K. oxytoca*, and *S*. Typhimurium are resistant to the bile component GCA when present in solid agar media, but that these bacteria are susceptible to this compound in a liquid culture, most likely due to changes in membrane permeability that has more pronounced effect in liquid medium. Additional Enterobacteriaceae species that were shown here to be susceptible for GCA in liquid culture are *R. ornithinolytica* and *C. freundii*. This phenotype leads to growth inhibition during the late logarithmic phase, before the culture enters the stationary phase. To the best of our knowledge, this is the first study showing that various Enterobacteriaceae family members are somewhat susceptible to a bile component in aqueous solution, but tolerate this condition while growing on a solid agar medium.

Using several independent biochemical approaches, we demonstrated that GCA increases permeability and compromises the integrity of Enterobacteriaceae bacterial membranes in liquid culture. Furthermore, clear differences in the membrane texture were identified using high-resolution scanning EM following delicate iridium coating, which reveals the fine details of the membrane surface ultrastructure. Previous reports have suggested that bile may cause membrane defects in mammalian cells and showed that cell exposure to bile salts can dissolve membrane lipids and cause dissociation of integral membrane proteins, leading to cell lysis (Coleman et al., 1980; Heuman et al., 1996). Other studies conducted with Gram-positive bacteria demonstrated that exposure of cells to bile resulted in a shrunken and altered surface morphology (De Valdez et al., 1997; Leverrier et al., 2003; Sannasiddappa et al., 2017), and indicated leakage of intracellular content, as demonstrated by enzymatic assays (Noh and Gilliland, 1993) and UV absorbance (Sannasiddappa et al., 2017). Although these studies have collectively suggested that bile modifies membrane permeability or integrity in Gram-positive bacteria, reports demonstrating membrane damage by bile in Gram-negative bacteria are scarce. The few published reports on bile-induced membrane damage in Gram-negative bacteria have mainly included indirect observations. For example, Fujisawa and Mori showed that the activity of β-glucuronidase in intact *E. coli* cells increased in the presence of bile salts, possibly due to higher substrate inflow (Fujisawa and Mori, 1996).

The finding that various Enterobacteriaceae species are resistant to GCA in solid media, but not in liquid culture is intriguing and challenges the current paradigm. These results also suggest that in the host, enteric bacteria are probably not protected from bile when they are planktonic, but only within biofilm communities. Indeed, many enteric bacterial species have been shown to enhance biofilm formation in response to bile salts or in the presence of bile, including *V. cholera* (Hung et al., 2006), *Shigella flexneri* (Nickerson et al., 2017), and *Salmonella* spp (Prouty et al., 2002; Crawford et al., 2008).

Of a special importance is the identified effect of GCA on bacterial conjugation, and consequently, the transfer frequency of antibiotic resistance genes. We show that 2% GCA can significantly inhibit bacterial conjugation and that this phenotype is associated with a prominent reduction in the observed occurrence of conjugative pili. Since transcriptional analysis has indicated that the transcription of multiple *tra* genes, involved in the expression and assembly of the conjugative pili was not changed, and also the expression of a tagged TraL, our current working hypothesis, is that GCA (but not necessarily other detergents, see **Supplementary Figure S2**) interferes with the assembly or integrity of the pilin subunits, composing the conjugative pili. This possibility is in agreement with a previous report suggesting that bile salts interfere with hydrophobic interfaces, causing protein complexes to dissociate and hydrophobic cores to be destabilized (Cremers et al., 2014).

In mammals, conjugated bile acids are actively excreted from liver hepatocytes into the bile and are stored in the gallbladder. After a meal, gallbladder contraction discharges bile acids into the small intestinal lumen, however when passing through the small intestine, most of the bile acids are reabsorbed and return to the liver, where they are resecreted into the bile (Ticho et al., 2019). After food ingestion, the physiological concentration of bile acids increases to ~12 mM (0.54% w/v) in the duodenum, but gradually descends down the ileum until reaching approximately 2 mM (0.08% w/v) in the terminal ileum due to their active absorption (Northfield and Mccoll, 1973). Therefore, the presented results may suggest that in the host, bacterial conjugation is inhibited at the proximal region of the small intestine, and that this inhibition is gradually relieved towards the distal region of the ileum and colon, where bile concentration is decreased. It is tempting to speculate that regulation of bacterial conjugation by bile has evolved to facilitate bacterial conjugation, specifically in the colon, where the microflora diversity, and thus the likelihood of fertile conjugation, is the highest, rather than in the duodenum, which is much less colonized by microorganisms.

Another effect of GCA on the physiology of Gram-negative bacteria is the enhancement of their sensitivity to antimicrobial agents. These results are consistent with a previous eminent report showing an increase in the antibiotic susceptibility of *S*. Typhimurium in the presence of different deconjugated bile acids (cholic acid and deoxycholic acid) (He et al., 2012). The reduced resistance to antibiotics in the presence of GCA is most likely due to the increase in membrane permeability, which enables a higher inflow of antibiotics into the bacterial cell via the compromised membrane in liquid media.

Despite the interesting findings reported in this study, a few limitations should be acknowledged. Because enteric bacteria are particularly known to be bile-resistant, members of this family were the focus of the study; however, the sensitivity of other Gram-negative bacteria was not tested. We expect that other Gram-negative bacteria that does not normally colonize the gut of mammalian hosts would demonstrate even higher susceptibility to bile and its components. Additional study limitation is that although we provide several independent evidences that GCA alters the structure and permeability of the Enterobacteriaceae cell membrane and reduces bacterial conjugation, the exact mechanism is not known and further chemo-physical characterization will be required to reveal how GCA interacts with and changes the membrane of Gram-negative bacteria. Another point that should be mentioned here is the effective concentration of GCA. We show that 2% of GCA are highly effective as conjugation and growth inhibitor and as an enhancer of antibiotic susceptibility. From pharmacological point of view, this is relatively high concentration and further optimization and structure relationship activity (SRA) studies are required for medical usage of this molecule.

## Conclusions

We show that the presence of the bile component GCA has a pleiotropic effect on the physiology of Enterobacteriaceae family members that are traditionally considered as relatively bile-resistant. We demonstrated that the growth of these bacteria is inhibited in liquid, but not in solid media, and that their membrane permeability and susceptibility to antibiotics are notably increasing in the presence of 2% GCA. Moreover, bacterial motility, conjugation frequency, and the occurrence of conjugative pili dramatically decreased following exposure to GCA. These results highlight the potential use of GCA as an antimicrobial agent that can fulfill a multidimensional purpose of limiting bacterial growth, preventing the conjugation and dissemination of antibiotic resistance genes, and increasing the antibiotic sensitivity of resistant bacteria. Further structural optimization and SRA studies may increase the effectiveness of this compound be used either alone or in combination with standard antibiotic therapy against resistant infections.

## Data Availability

The original contributions presented in the study are included in the article/**Supplementary Material**. Further inquiries can be directed to the corresponding author/s.
