# Supplementary material for: The Effect of glycocholic acid on the growth, membrane permeability, conjugation and antibiotic susceptibility of Enterobacteriaceae
Source: Front Cell Infect Microbiol. 2025 Mar 20;15:1550545. doi: 10.3389/fcimb.2025.1550545 (PMC12006743; doi:10.3389/fcimb.2025.1550545)
Supplement: Supplementary file 1 [file Table1.docx]

**Supplementary Information**

**The effect of mating time on GCA conjugation inhibition.**

To verify that conjugation for 6 h does not "saturate" bacterial mating in a way that masks the inhibitory effect of GCA on conjugation, bacterial mating between *E. coli* K1037 harboring pN3 (Tc^R^; donor) and *E. coli* K-12 ORN 172 (Km^R^; recipient) was evaluated on LB-agar plates and LB-agar supplemented with 2% (W/V) GCA for 1, 2, 3, and 6 h. These varying mating times demonstrated reduced conjugation frequencies by 8, 10, 11, and 12.5-fold, respectively (Fig. S1), indicating a time-response effect and increasing conjugation inhibition with time.

**GCA reduces the prevalence of drug-resistant Gram-negative bacteria in the environment**.

Building on the observations presented in the main text, we have sought to test whether GCA could decrease the prevalence of antibiotic-resistant communities in the environment. Previous studies have demonstrated that the piping systems and sewage spillways of hospitals are hotspot reservoir for antibiotic resistant bacterial populations (D'Alessandro et al., 2016;Regev-Yochay et al., 2018;Salazar et al., 2022). To experimentally examine the effect of GCA on the prevalence of antibiotic resistant strains in the nosocomial environment, we have used kitchen cellulose sponges, socked with either saline (control) or saline supplemented with 1% GCA (experiment). These sponges were immobilized approximately 10 cm above the bottom of five independent sewage pits in the central sewage pipeline of the Sheba Medical Center, in a way that they were exposed to the passing fluids, but didn't washed out (Fig. S5 A-B). Three or six experimental and control sponges were collected from each pit in two independent experiments that lasted 20 days and in a third experiment that lasted 39 days.

After the sponges were collected (60 sponges of the experiment and 60 sponges of the control were collected and analyzed in total), they were vigorously washed with saline to extract adherent bacteria and the concentrated wash flow was plated on MacConkey agar plates to select for Enterobacteriaceae isolates. Sponges-recovered colonies were picked up and transferred to CHROMagar orientation PD-165 plates and to MacConkey agar plates supplemented with chloramphenicol to determine the prevalence of chloramphenicol-resistant bacteria. Testing chloramphenicol resistance was specifically addressed because in a preliminary screen, high prevalence of chloramphenicol resistance was identified among the sewage Enterobacteriaceae populations at the Sheba Medical Center (data not shown). Overall, during these three experiments, chloramphenicol resistance was tested for 485 Enterobacteriaceae isolates that were recovered from LB containing sponges and from 444 CFUs recovered from GCA containing sponges. Interestingly, both after 20 days (Fig. S5C) and after 39 days (Fig. S5D), significantly lower proportion of chloramphenicol-resistant bacteria were isolated from sponges that contained saline supplemented with GCA than from sponges that were socked with saline only. To further examine these results, we have randomly chosen 12 Enterobacteriaceae isolates and tested their growth in LB in the presence of chloramphenicol or GCA. As shown in Fig. S6, all of the tested isolates presented impaired growth in the presence of GCA. Altogether, these results suggest that GCA can reduce the burden of antibiotic resistant bacteria circulating in wet environments such as the sewage.

**Supplementary figures**

**Fig. S1. The effect of GCA on bacterial conjugation under varying mating times.** Plate mating assays between *E. coli* K1037 harboring pN3 (Tc^R^; donor) and *E. coli* K-12 ORN 172 (Km^R^; recipient) were conducted on LB-agar plates and on LB-agar supplemented with 2% GCA at 37°C for 1, 2, 3, and 6 h. Conjugation frequency was calculated as the number of obtained transconjugants/ number of donor CFUs. The graph shows the mean and the standard error of the mean (SEM) of three independent experiments. Unpaired t-test was used to determined statistical significance. ***, p <0.001.

**Fig. S2. Inhibition of bacterial conjugation is not a general detergents trait. (A** and **C)** Plate mating assays between *E. coli* K1037 harboring pN3 (Tc^R^; donor) and *E. coli* K-12 ORN 172 (Km^R^; recipient) were conducted on 0.45 µm filters (Sartorius) placed onto LB agar plates, LB agar plates supplemented with 0.5 and 2 % (W/V) GCA **(A)** or LB agar plates supplemented with 0.5 or 2 % (V/V) Triton X-100 **(C)** at 37°C for 6 h. Conjugation frequency was calculated as the number of obtained transconjugants CFUs/ number of donor CFUs and is shown relative to the conjugation frequency on LB-agar, which was normalized to 100%. The charts show the mean and the standard error of the mean (SEM) of three independent biological experiments. One-way ANOVA against the conjugation frequency on LB was used to determined statistical significance. ns, not significant, ***, p <0.001. (**B** and **D**) CFUs count of the donor strain (*E. coli* K1037 harboring pN3) following 6 h incubation on 0.45 µm filter on LB agar plates, LB agar plates supplemented with 0.5 or 2 % (W/V) GCA **(B)**, or 0.5 or 2 % (V/V) Triton X-100 **(D)** that was conducted in parallel to the mating experiments**.** The charts show the mean and SEM of 3 independent biological repeats. One-way ANOVA against the CFUs count on LB was used to determined statistical significance. ns, not significant.

**Fig. S3. The effect of GCA on EtBr fluorescence.** Ethidium bromide (EtBr) was added at a final concentration of 20 µM into PBS (black line), or PBS supplemented with 0.5% (green line), 1% (blue line) or 2% (red line) GCA sodium salt. One µg genomic DNA from *E. coli* K1037 was added to all samples and the fluorescence intensity (excitation 520 nm, emission 600 nm) was measured every two minutes. The change in fluorescence intensity is presented relative to the fluorescence of EtBr in PBS at T0, which was set to 100%. The chart presents the mean and SEM of six replicates.

**Fig. S4. GCA changes the morphology of bacterial cells.** A stationary phase culture of *E. coli* K1037 was subcultured into fresh LB broth (**A**) or LB broth supplemented with 2% glycocholic acid (**B**) for 6 h at 37°C. 100 µL from the liquid subcultures were added to 250 µL phosphate-buffered saline (PBS) and imaged using a Merlin scanning electron microscope (Zeiss), after sputter iridium coating. Representative images of *E. coli* cells at different cell division stages are shown.

**Figure S5. Glycolic acid reduces the burden of antibiotic resistant strains in the environment. (A and B)** groups of 6 cellulose kitchen sponges socked with saline (yellow) or saline supplemented 1% GCA (magenta) were immobilized with flexible strings and positioned approximately 10 cm above the bottom of five independent sewage pits of the Sheba Medical Center central sewage pipeline. After 20 **(C)** or 39 days **(D)** the sponges were collected and the extracted bacteria were plated on MacConkey agar plates that were incubated for 16 h at 37°C. Growing colonies were picked up and replica plated onto fresh MacConkey agar, CHROMagar orientation PD-165, and MacConkey agar supplemented with chloramphenicol (25 μg /ml) plates. The proportion of chloramphenicol resistance among bacteria that were recovered from the sponges socked with and without GCA was calculated for each sewage pit. Unpaired t test (for C) or Z-test for independent proportions (for D) was used to determined statistical significance. *, p<0.05; ***, p < 0. 001.

**Figure S6. GCA limits the growth of antibiotic resistant environmental Enterobacteriaceae isolates.** The growth of 12 environmental Enterobacteriaceae isolates, which were obtained from the sewage of the Sheba Medical Center was tested in LB broth (black curves), LB supplemented with 25 μg /ml chloramphenicol (blue curves) and LB supplemented with 2 % GCA (red curves). Optical density (600 nm) was measured every 30 minutes, during 20 h incubation at 37°C of **(A)** *Raoultella ornithinolytica* SMCSS-1, **(B)** *Klebsiella oxytoca* SMCSS-2, **(C)** *Raoultella ornithinolytica* SMCSS-3, **(D)** *Citrobacter werkmanii* SMCSS-4, **(E)** *Citrobacter freundii* SMCSS-5, **(F)** *Citrobacter freundii* SMCSS-6, **(G)** *Citrobacter freundii* SMCSS-7, **(H)** *Raoultella ornithinolytica* SMCSS-8, (I) *Citrobacter freundii* SMCSS-9, **(J)** *Citrobacter freundii* SMCSS-10, **(K)** *Klebsiella oxytoca* SMCSS-11, and **(L)** *Klebsiella oxytoca* SMCSS-12. The graphs present one representative experiment with 3 biological repeats. Two-way ANOVA was used to determined statistical significance. ***, p<0.001.

**Supplementary Tables**

**Table S1. Bacterial strains and plasmids used in this study**

| **Strain name** | **Description** | **Source or reference** |
| --- | --- | --- |
| *E. coli* K-12 ORN172 | A recipient strain for conjugation (∆*fim*BEACDFGH), Km^R^ | (Woodall et al., 1993) |
| *E. coli*  K1037/ pN3 | *E. coli*  K1037 harboring incN plasmid N3 | Coli Genetic Stock Center  CGSC 7793 |
| *E. coli* J5-3 | A recipient strain for conjugation, Rifampicin^R^ | Noemi Nogrady lab |
| *E. coli* 16283 | *E. coli* isolated from a urinary tract infection patient | Sheba Medical Center |
| *Salmonella enterica* Typhimurium (SL1344) | *Salmonella enterica* serovar Typhimurium (SL1344) | - Lab collection |
| *Klebsiella pneumonia* MGH78578 | *Klebsiella pneumoniae* MGH78578 subsp. *Pneumonia* ATCC 700721 | Michael McClelland lab |
| *Klebsiella huaxiensis* -SMCSS p2C1 | *Klebsiella huaxiensis* environmental isolate from the Sheba Medical Center sewage system harboring 2C1 plasmid | This study |
| *Klebsiella oxytoca* SMCSS-p7T2 | *Klebsiella oxytoca* environmental isolate from Sheba Medical Center sewage system harboring 7T2 plasmid | This study |
| *Klebsiella oxytoca* SMCSS-2 | environmental isolate from Sheba medical center sewage system | This study |
| *Klebsiella oxytoca* SMCSS-11 | environmental isolate from Sheba medical center sewage system | This study |
| *Klebsiella oxytoca* SMCSS-12 | environmental isolate from Sheba medical center sewage system | This study |
| *Raoultella ornithinolytica* SMCSS-1 | environmental isolate from Sheba medical center sewage system | This study |
| *Raoultella ornithinolytica* SMCSS-8 | environmental isolate from Sheba medical center sewage system | This study |
| *Raoultella ornithinolytica* SMCSS-3 | environmental isolate from Sheba medical center sewage system | This study |
| *Citrobacter werkmanii* SMCSS-4 | environmental isolate from Sheba medical center sewage system | This study |
| *Citrobacter freundii* SMCSS-5 | environmental isolate from Sheba medical center sewage system | This study |
| *Citrobacter freundii* SMCSS-6 | environmental isolate from Sheba medical center sewage system | This study |
| *Citrobacter freundii* SMCSS-7 | environmental isolate from Sheba medical center sewage system | This study |
| *Citrobacter freundii* SMCSS-9 | environmental isolate from Sheba medical center sewage system | This study |
| *Citrobacter freundii* SMCSS-10 | environmental isolate from Sheba medical center sewage system | This study |
| *Staphylococcus aureus* ATCC 25923 | A whole-genome sequenced clinical isolate used as a standard laboratory testing control strain | ATCC |
|  | | |
| **Plasmid name** | **Description** | **Source** |
| pN3 | incN conjugative plasmid N3 | *Coli* Genetic Stock Center  CGSC 7793 |
| pRP4 | incP-α conjugative plasmid RP4 | *Coli* Genetic Stock Center  CGSC 12352 |
| pRK2 | incP conjugative plasmid RK2 | *Coli* Genetic Stock Center  CGSC 12353 |
| pCVM29188 | incI conjugative plasmid CVM29188 | (Fricke et al., 2009) |
| pESI | 285 kb conjugative plasmid | (Aviv et al., 2014) |
| p2C1 | Conjugative plasmid of *Klebsiella huaxiensis* -SMCSS p2C1 | This study |
| p7T2 | Conjugative plasmid of *Klebsiella oxytoca* SMCSS-p7T2 | This study |
| pWSK29 TraL-2HA | pWSK29 with 2HA tagged *traL* under its native promoter | (Piscon et al., 2023) |

**Table S2. Primers used in this study**

| **Primer name** | **Sequence 5'-3'** | **Description** |
| --- | --- | --- |
| traG F | GGGATCAGGGGCGTTATCTG | qRT-PCR |
| traG R | CTGAAAATCCCCTCGCTGGT | qRT-PCR |
| traC F | GATCTACAGCGACGCCATGA | qRT-PCR |
| traC R | CGCCTTTTTCAGCCAGCTTT | qRT-PCR |
| traL F | CTGGGTCTTTCGGTTGACGA | qRT-PCR |
| traL R | GTGAGTGAGCCGGTGTTGTA | qRT-PCR |
| traK F | CGGGAGTCCGGGTTGAAAAT | qRT-PCR |
| traK R | TACGCCAGATAGCGTTTCGG | qRT-PCR |
| traI F | CGCGACAGCCGTTATTTCAG | qRT-PCR |
| traI R | AACCGTCAATTTGTTGCCCG | qRT-PCR |
| traJ F | ATCCGATCCTCAGAAGCCCT | qRT-PCR |
| traJ R | TTGAACTTAGCGACGGGCAT | qRT-PCR |
| fliC F | GAACAGGTGTACCGCCTGAA | qRT-PCR |
| fliC R | AGTGACAATGGCGACTGGAG | qRT-PCR |
| 16S F | GGTTAAGTCCCGCAACGAG | qRT-PCR |
| 16S R | CTTCTCTTTGTATGCGCCATTG | qRT-PCR |
| 8F_16s_rRNA | AGAGTTTGATCCTGGCTCAG | 16S rRNA sequencing |
| 518R_16s_rRNA | GTATTACCGCGGCTGCTGG | 16S rRNA sequencing |
| traL+prom F | TTTTGAGCTCTCGTTTTTCTGATTTCAGGGGAA | TraL-2HA cloning |
| traL+prom R | AAAATCTAGATTCCCCCTTCGCTGTTTCCT | TraL-2HA cloning |

**SUPPLEMENTARY References**

Aviv, G., Tsyba, K., Steck, N., Salmon-Divon, M., Cornelius, A., Rahav, G., Grassl, G.A., and Gal-Mor, O. (2014). A unique megaplasmid contributes to stress tolerance and pathogenicity of an emergent Salmonella enterica serovar Infantis strain. *Environ Microbiol* 16**,** 977-994.

D'alessandro, D., Nusca, A., and Napoli, C. (2016). Are liquids an efficient vehicle of healthcare associated infections? A review of reported cases in Italy (2000-2014). *Ann Ig* 28**,** 416-431.

Fricke, W.F., Mcdermott, P.F., Mammel, M.K., Zhao, S., Johnson, T.J., Rasko, D.A., Fedorka-Cray, P.J., Pedroso, A., Whichard, J.M., Leclerc, J.E., White, D.G., Cebula, T.A., and Ravel, J. (2009). Antimicrobial resistance-conferring plasmids with similarity to virulence plasmids from avian pathogenic Escherichia coli strains in Salmonella enterica serovar Kentucky isolates from poultry. *Appl Environ Microbiol* 75**,** 5963-5971.

Piscon, B., Pia Esposito, E., Fichtman, B., Samburski, G., Efremushkin, L., Amselem, S., Harel, A., Rahav, G., Zarrilli, R., and Gal-Mor, O. (2023). The Effect of Outer Space and Other Environmental Cues on Bacterial Conjugation. *Microbiol Spectr* 11**,** e0368822.

Regev-Yochay, G., Smollan, G., Tal, I., Pinas Zade, N., Haviv, Y., Nudelman, V., Gal-Mor, O., Jaber, H., Zimlichman, E., Keller, N., and Rahav, G. (2018). Sink traps as the source of transmission of OXA-48-producing Serratia marcescens in an intensive care unit. *Infect Control Hosp Epidemiol* 39**,** 1307-1315.

Salazar, C., Gimenez, M., Riera, N., Parada, A., Puig, J., Galiana, A., Grill, F., Vieytes, M., Mason, C.E., Antelo, V., D'alessandro, B., Risso, J., and Iraola, G. (2022). Human microbiota drives hospital-associated antimicrobial resistance dissemination in the urban environment and mirrors patient case rates. *Microbiome* 10**,** 208.

Woodall, L.D., Russell, P.W., Harris, S.L., and Orndorff, P.E. (1993). Rapid, synchronous, and stable induction of type 1 piliation in Escherichia coli by using a chromosomal lacUV5 promoter. *J Bacteriol* 175**,** 2770-2778.
