# Supplementary figures and images for: The Effect of glycocholic acid on the growth, membrane permeability, conjugation and antibiotic susceptibility of Enterobacteriaceae
Source: Front Cell Infect Microbiol. 2025 Mar 20;15:1550545. doi: 10.3389/fcimb.2025.1550545 (PMC12006743; doi:10.3389/fcimb.2025.1550545)

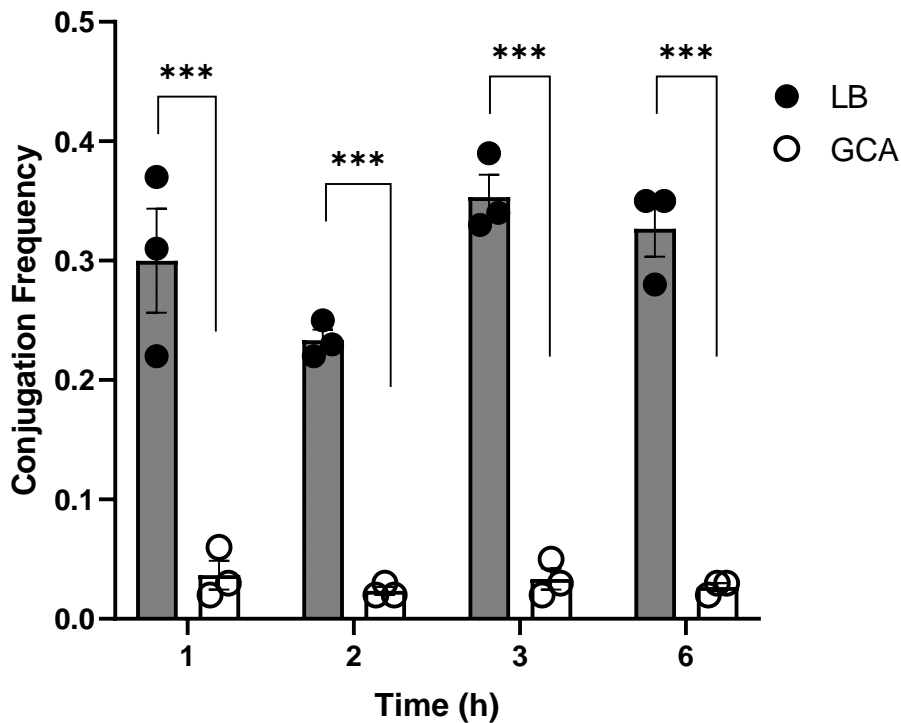

**Fig. S1**

Supplement: Supplementary file 2 [file Image1.pdf]

**A**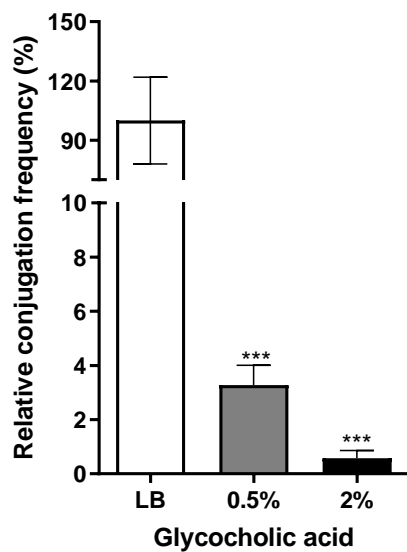**B**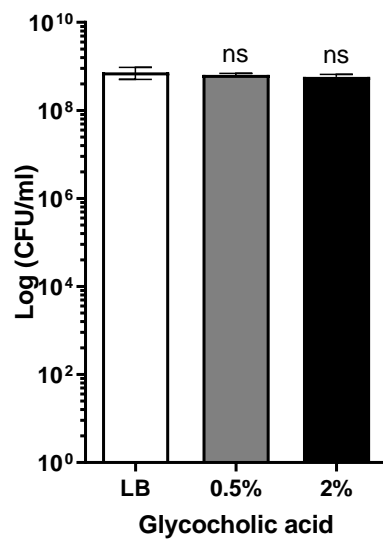**C**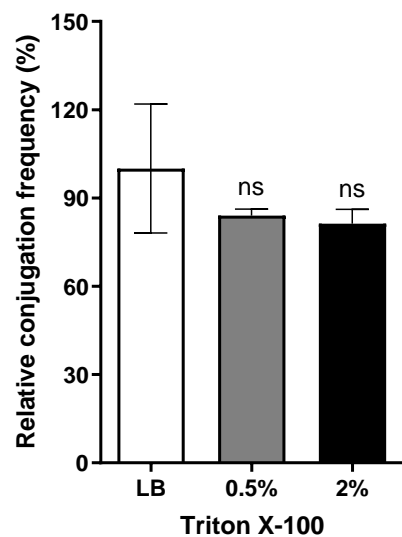**D**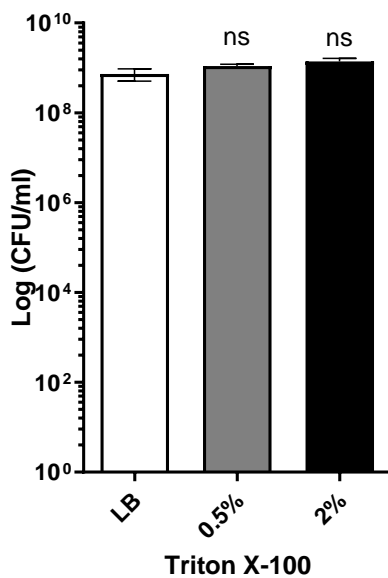**Fig. S2**

Supplement: Supplementary file 3 [file Image2.pdf]

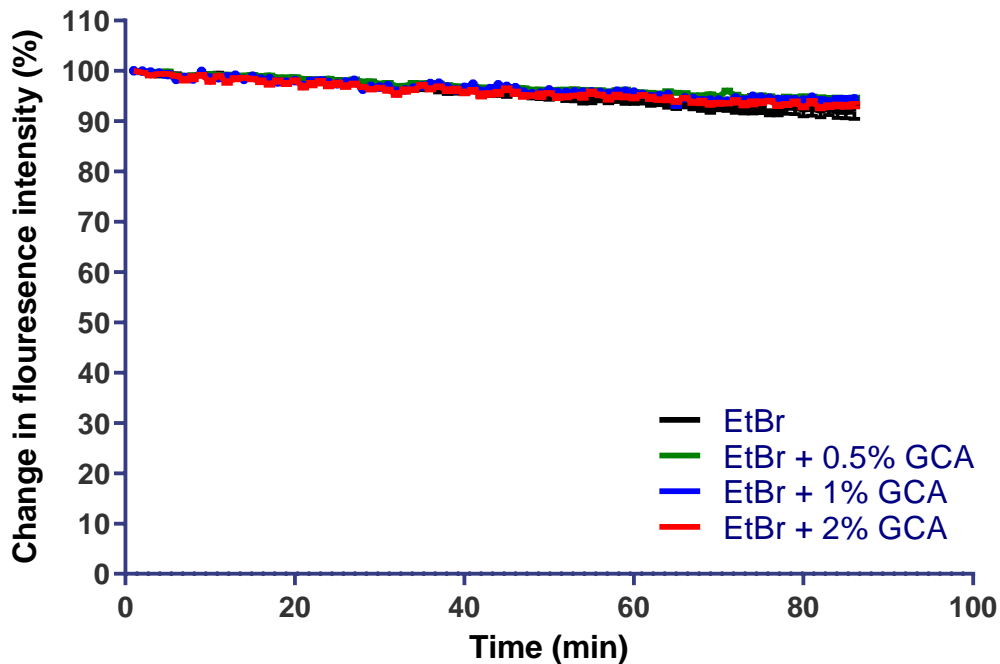

**Fig. S3**

Supplement: Supplementary file 4 [file Image3.pdf]

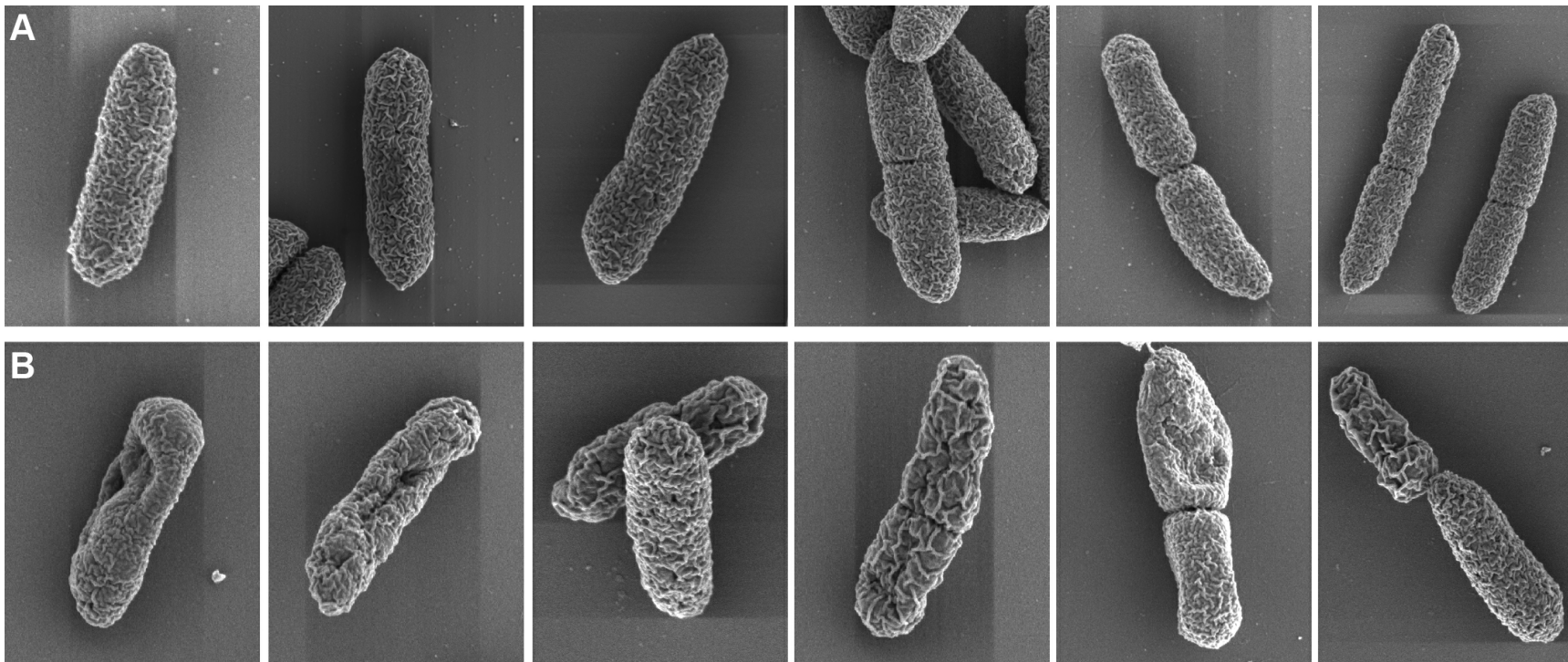

**Fig. S4**

Supplement: Supplementary file 5 [file Image4.pdf]

**A**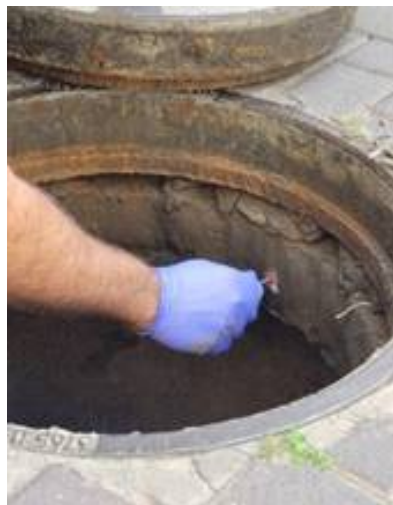**B**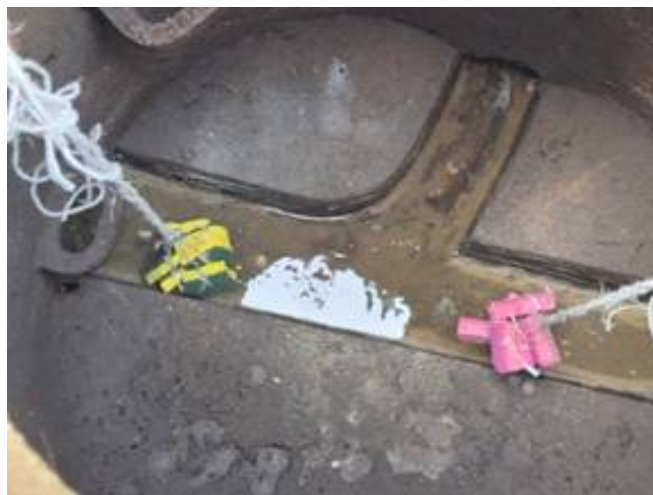**C**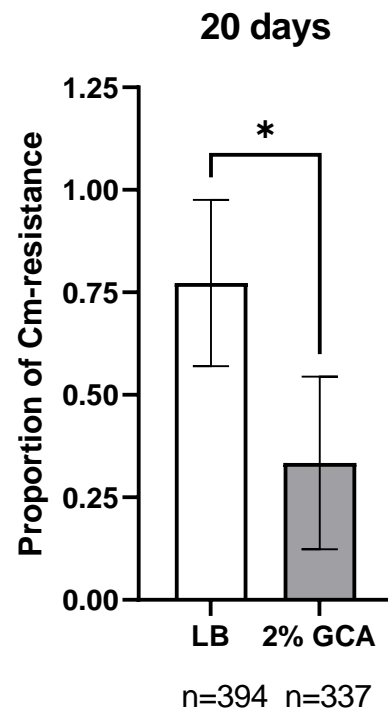**D**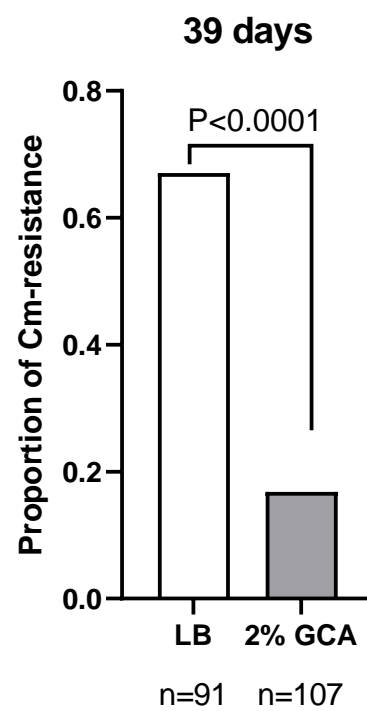**Fig. S5**

Supplement: Supplementary file 6 [file Image5.pdf]

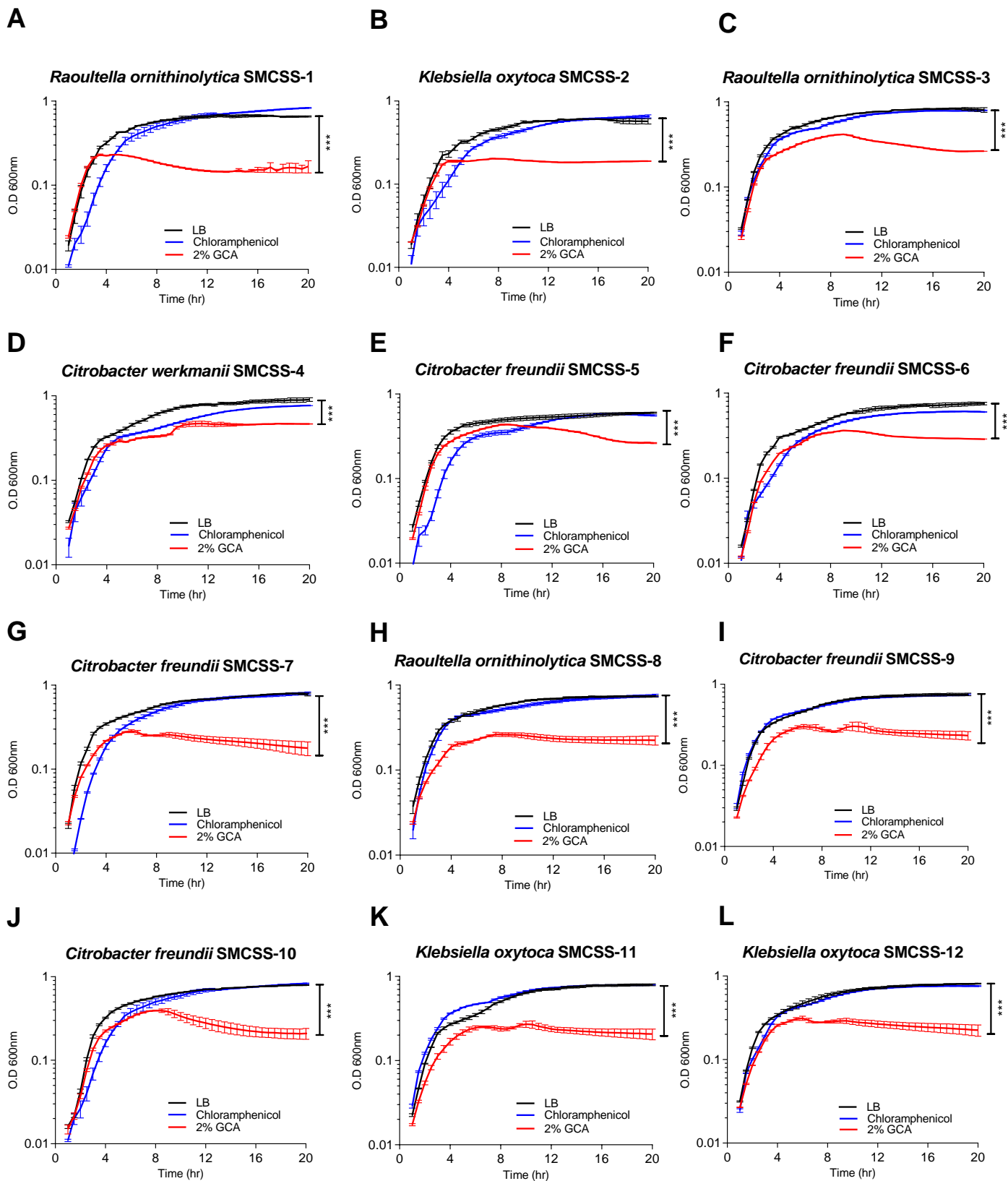

**Fig. S6**

Supplement: Supplementary file 7 [file Image6.pdf]
